# Research progress on the prevention and treatment of zearalenone poisoning in animals using natural products

**DOI:** 10.3389/fvets.2025.1710151

**Published:** 2025-11-21

**Authors:** Nannan Liu, Qi Zhang, Yulan Piao, Chenghe Sun, Guangliang Shi

**Affiliations:** 1 Jilin Agricultural Science and Technology College, Jilin, China; 2 College of Veterinary Medicine, Northeast Agricultural University, Harbin, China; 3 Heilongjiang Provincial Key Laboratory of Pathogenic Mechanism for Animal Disease and Comparative Medicine, Harbin, China; 4 Institute of Chinese Veterinary Medicine, Northeast Agricultural University, Harbin, China

**Keywords:** zearalenone, toxicity, natural products, detoxification methods, bee pollen

## Abstract

Zearalenone (ZEA) is a non-steroidal estrogenic mycotoxin produced by Fusarium fungi, widely present in cereal feeds such as corn, barley, wheat, and sorghum. It not only impacts agricultural production and feed safety but also poses a serious threat to animal health. Extensive research demonstrates that natural products can effectively mitigate the toxic effects of zearalenone. This paper reviews zearalenone’s physicochemical properties and toxicological effects, with a focus on advances in the research on reducing zearalenone toxicity through plant, microbial, and mineral-derived natural products. The aim is to provide theoretical references for developing more efficient and safer zearalenone detoxification agents.

## 1 Introduction

Zearalenone (ZEA), also known as F-2 toxin, is a non-steroidal estrogenic mycotoxin produced by Fusarium graminearum fungi. It is one of the top three mycotoxins in animal feed globally (1). Grain feeds such as wheat and corn are susceptible to contamination by zearalenone and other toxins during production, processing, and transportation. Furthermore, ZEA exhibits chemical stability, produces numerous metabolites, and persists for extended periods. The Food and Agriculture Organization of the United Nations (FAO) estimates that approximately 25% of the world’s grain production is contaminated with mycotoxins (2). Based on ZEA monitoring data from China between 2004 and 2024, Wang et al. (3) Comprehensively analyzed ZEA contamination levels in Chinese feed and raw materials. International comparisons indicate that China’s zearalenone contamination levels exceed those in Europe.

The phenol-dihydroxy lactone structure of ZEA resembles that of estrogen. When animals consume ZEA-contaminated grain feed, it undergoes metabolic conversion within the body and is transported via the bloodstream throughout the system. There, it binds to estrogen receptors, triggering various pathological processes including estrogenic effects, oxidative stress, apoptosis, and inflammation. This leads to multi-organ damage affecting the uterus, testes, liver, kidneys, and spleen. Affected animals exhibit symptoms including prolonged estrus cycles, miscarriages, testicular atrophy, and poor sperm quality (4–7), causing substantial economic losses in livestock farming and feed processing industries. Currently, ZEA detoxification methods face limitations such as low efficacy, high costs, and significant toxicity (8–10).

In recent years, extensive research has focused on the antagonistic effects of natural plant extracts, microorganisms and their metabolites, and mineral materials against ZEA. Natural products, with their advantages of high safety, wide availability, and diverse mechanisms of action, have gradually become a hotspot in ZEA detoxification research. For example, a variety of bioactive substances found in natural plants, such as flavonoids, polysaccharides, and polyphenols, can successfully lessen the harm that ZEA does to organs, including the kidneys, liver, testes, and uterus. This is accomplished by scavenging reactive oxygen species, blocking inflammatory and apoptotic signaling pathways, and counteracting the estrogen-like actions of ZEA (11–14). This review systematically outlines the physicochemical properties, toxicity mechanisms, and mitigation methods of ZEA, with a focus on summarizing research progress in natural medicines for zearalenone mitigation. It aims to provide a theoretical foundation for the innovation of ZEA pollution prevention and control technologies and the industrial application of natural mitigants, thereby contributing to the improvement of food security and public health safeguards.

## 2 Zearalenone

### 2.1 Physicochemical properties of zearalenone

**Figure 1:**
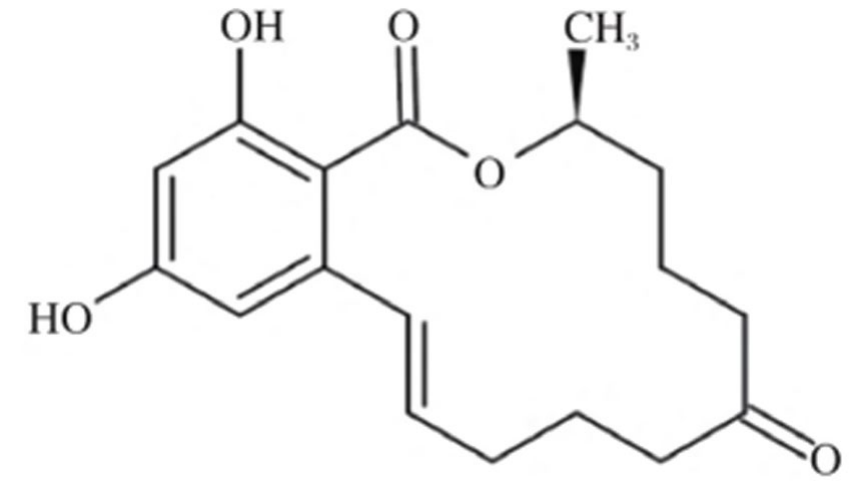
Chemical formula of zearalenone in corn. Reproduced with permission from Wu et al. (15).

ZEA, chemically named 6-(10-hydroxy-6-oxo-undec-2-enyl)*β*-resorcylic acid lactone, has the molecular formula C₁₈H₂₂O₅. Its molecule contains two phenolic hydroxyl groups and one lactone ring, belonging to the *β*-resorcylic acid lactone class of compounds. Its chemical structure is shown in Figure 1 (15). ZEA exhibits stable chemical properties with a well-defined melting point. The pure compound melts at 164–165 °C and gradually decomposes when heated above 200 °C. Its toxicity is difficult to destroy through high-temperature treatment. At room temperature, its structure remains stable. Although hydrolysis occurs under alkaline conditions, its structure can be restored by lowering the pH. ZEA is readily soluble in polar organic solvents such as methanol, ethanol, acetone, and chloroform, but poorly soluble in water (Table 1).

**Table 1:** Mitigating effects of natural products on ZEA and their applications.

Types of natural products	Representative substance	Primary mechanism of action	Experimental model/effect(s)	References
Plant-derived Flavonoids	Rutin, Quercetin, Soy Isoflavones	Antioxidant: Scavenges ROS and enhances SOD and GSH-Px activity. Anti-apoptotic/Anti-inflammatory: Regulates Bax/Bcl-2 balance and inhibits NF-κB pathway. Anti-endocrine disruption: Competitively binds to estrogen receptors.	Alleviating ZEA damage to porcine endometrial cells, mouse liver, and male reproductive systems.	(44–58)
Plant-derived polysaccharides	Astragalus polysaccharides, nettle polysaccharides	1. Antioxidant Effects: Enhances SOD and GSH-Px activity while reducing MDA levels. 2. Immunomodulation: Inhibits ERS and apoptosis pathways (Bax/Caspase-3). 3. Signal Pathway Regulation: Involves pathways such as PI3K-Akt and glutathione metabolism.	Protects porcine testicular supporting cells and avian thymus cells, alleviating oxidative damage to the liver and kidneys.	(57–63)
Plant-derived Polyphenols	Proanthocyanidins, Curcumin, Resveratrol	1. Antioxidant: Activates the Nrf2/ARE pathway, upregulating the expression of HO-1 and other genes. 2. Anti-inflammatory: Inhibits NLRP3 inflammasome and NF-κB pathways. 3. Regulates the microbiota-organ axis: For example, curcumin modulates the “gut microbiota-testicular axis.”	Reduce the toxicity of ZEA in mouse intestinal, liver, and testicular tissues, as well as in porcine kidney cells.	(62–70)
Microbial Source	*Bacillus cereus*, Meyerozyma caribbica, Lactic Acid Bacteria Consortium (LBC-4)	1. Secreting Degradative Enzymes: For instance, Bacillus belerensis produces CotA laccase and Prx peroxidase reductase, efficiently degrading ZEA into low-toxicity products under high-temperature, alkaline conditions. 2. Adsorption and biodegradation: For example, functional groups on the cell surface of Meyerozyma caribbica adsorb ZEA, while intracellular enzymes (laccase, peroxidase) biotransform it. The lactic acid bacteria Consortium (LBC-4) can degrade ZEA into less toxic derivatives.	1. Bacillus baileyensis exhibits an in vitro degradation rate of over 91% for ZEA, with significantly reduced estrogenic activity in the degradation products. 2. At a concentration of 1 × 10^8^ cells/mL, Meyerozyma caribbica achieves an 80.3% degradation rate for ZEA. 3. The lactic acid bacteria Consortium (LBC-4) exhibits optimal attenuation effects at 37 °C and pH 7–8.	(70–78)
Mineral Source	Montmorillonite, Activated Carbon	Physical adsorption: Adsorption of ZEA molecules through charge interactions and pore structure.	Adsorption efficiency is limited, and desorption occurs; it may indiscriminately adsorb nutrients, resulting in restricted applications.	(78–81)
Bee-derived Products	Honey, Bee Pollen, Propolis	Antioxidant, Anti-inflammatory, Immunomodulatory: Rich in polyphenols, flavonoids; enhances antioxidant enzymes (SOD, CAT, GSH-Px); modulates immune function.	Honey: protects against liver injury, improves semen quality. Bee Pollen/Propolis: enhances total antioxidant capacity, immune cell activity, and antibody production in broilers.	(81–84)

### 2.2 Toxic effects of zearalenone

#### 2.2.1 Reproductive toxicity

**Figure 2:**
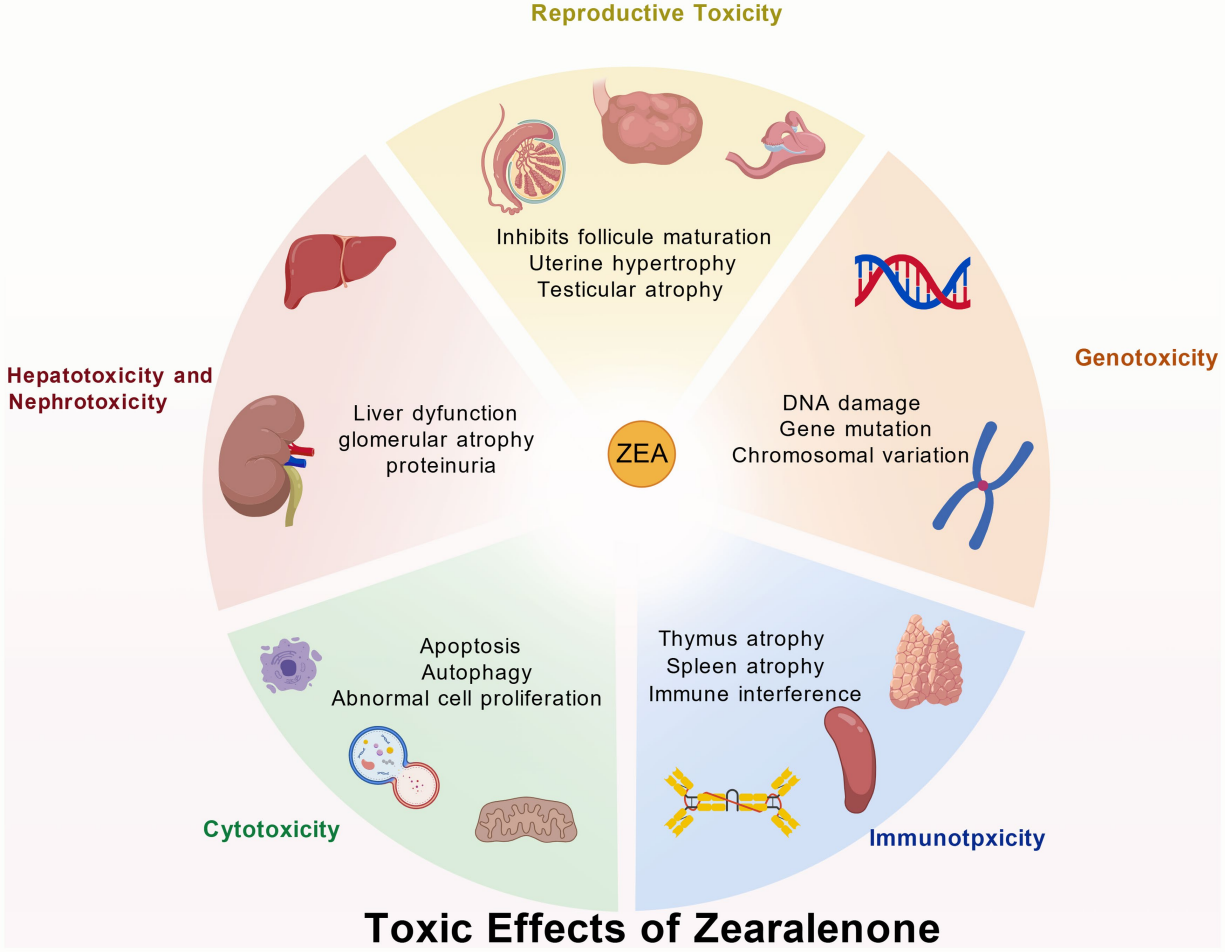
Toxic effects of zearalenone. Created with BioGDP.com.

The reproductive organs are the primary target sites for ZEA toxicity. ZEA, structurally similar to estrogen, can competitively bind to estrogen receptors within the body, thereby disrupting the normal synthesis of steroid hormones such as estradiol, testosterone, and progesterone (16). When sows ingest ZEA-contaminated feed, it readily damages reproductive organs such as the uterus and ovaries, manifesting clinical symptoms including vulvar swelling, mammary gland redness and swelling, estrus cycle disruption, and abortion (17). Gilt sows are particularly sensitive to ZEA; estrogen poisoning can occur when feed contains ZEA levels exceeding 1 mg/kg (18). Research proved that ZEA can cause abnormal follicular development in laying hens, characterized by increased stromal cell numbers, vacuolation-induced edema, accompanied by congestion, hemorrhage, oocyte retraction, and separation of the granulosa and theca membranes (19, 20). At concentrations exceeding 5 mg/kg, ZEA leads to reduced egg production, heightened inflammation, impaired ovarian function, and disrupted sex hormone secretion (21). ZEA activates the phosphorylation of AMPK in endometrial epithelial cells. This process modulates TSC2 and Rheb, affecting mTOR phosphorylation levels and subsequently inducing autophagy. Concurrently, it upregulates the expression of proliferative genes PCNA and BCl_2_ while downregulating the apoptotic gene Bax. After promoting proliferation of endometrial epithelial cells, this ultimately leads to thickening of the endometrium and myometrium, increased density of uterine glands, and induces uterine hypertrophy (22) (Table 2; Figure 2).

**Table 2:** Mechanisms and application prospects of natural products in countering ZEA toxicity.

Action dimension	Core strategy	Representative natural products	Specific mechanisms and targets	Advantages	Limitations and challenges
Source Elimination	Biological Degradation	*Bacillus velezensis*, B*acillus amyloliquefaciens*	Secretes enzymes (e.g., CotA laccase, Prx peroxiredoxin) to directly break down ZEA into low-toxicity products (e.g., C17H24O4)	Efficient and specific; removes the toxin at its root; functions under broad conditions (thermostable, alkali-tolerant)	Environmental stability of strains/enzymes; long-term safety of degradation products requires comprehensive evaluation; industrial production costs
*In Vivo* Antagonism	Receptor Competition	Soy Isoflavones	Competitively binds to estrogen receptors (ERα/ERβ), blocking ZEA’s estrogen-like effects	Direct mechanism; significant protective effects on the reproductive system; additional nutritional benefits	Efficacy depends on dose ratio; may produce complex estrogenic regulatory effects at high doses
	Signaling Pathway Regulation	Curcumin, Resveratrol, Proanthocyanidins, *Potentilla anserina* Polysaccharide	Activates antioxidant pathways (e.g., Nrf2/ARE, SIRT1/FOXO1); inhibits inflammation/apoptosis pathways (e.g., NF-κB, JNK)	Multi-target, comprehensive benefits; simultaneously alleviates oxidative stress, inflammation, and apoptosis	Complex composition makes mechanism studies difficult; potentially low absorption and bioavailability
Physical Protection	Adsorption	Montmorillonite, Activated Carbon	Non-specific physical adsorption via layered structure and high surface area, reducing intestinal absorption of ZEA	Low cost; easy to use; suitable for preliminary management of acute poisoning	Low adsorption efficiency for ZEA; risk of desorption; non-selectively adsorbs nutrients (vitamins, minerals)
Systemic Repair	Antioxidation/Immunoregulation	Astragalus Polysaccharide, Honey, Bee Pollen, Propolis	Enhances activity of endogenous antioxidant enzymes (e.g., SOD, GSH-Px); protects immune organs (thymus, spleen); modulates cytokine secretion	Boosts the body’s innate resistance; multiple functions (nutrition, antioxidation, immunity); high safety profile	Relatively slow onset; effects are indirect; quality and source of bee products can vary greatly
Ecological Intervention	Gut Microbiota Modulation	Lactic Acid Bacteria, Curcumin	Maintains gut microbiota balance (e.g., increases Lactobacillus abundance); repairs intestinal barrier; may confer indirect protection via the “gut-liver axis” or “gut-testis axis”	Focuses on holistic health; may reduce intestinal absorption and reabsorption of ZEA	Mechanistic research is still in early stages; effects show individual variability

**Figure 3:**
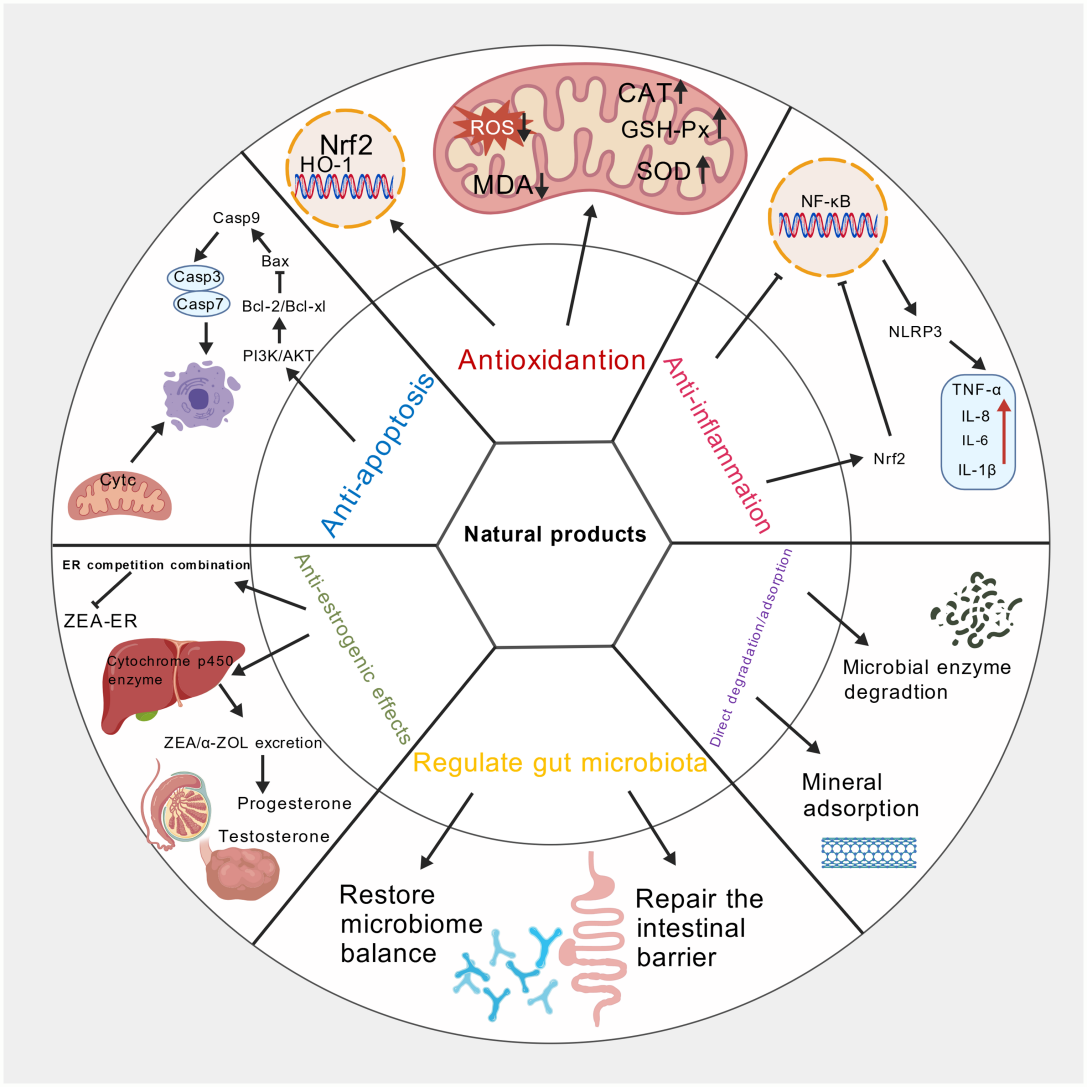
Mechanism diagram of natural products alleviating zearalenone toxicity. Created with BioGDP.com.

ZEA poisoning can also cause male animals to exhibit symptoms such as decreased sperm production and quality. Testicular interstitial cells are the primary site for testosterone synthesis in males. Studies indicate that ZEA significantly damages mitochondria in porcine testicular interstitial cells, inducing apoptosis through the PI3K-AKT signaling pathway mediated by mitochondria, which regulates the Bcl-2 protein family (23). ZEA induces mitochondrial morphological abnormalities and functional disorders, leading to mtDNA leakage into the cytoplasm. Free mtDNA acts as a damage-associated molecular pattern (DAMP), activating the endoplasmic reticulum transmembrane protein STING, which in turn triggers the NF-κB signaling pathway. This pathway not only upregulates inflammatory factor expression but also induces pyroptosis by regulating NLRP3 inflammasomes, resulting in damage to testicular and mouse testicular supporting cells (24). In both *in vivo* and *in vitro* studies of ZEA-induced male goat reproductive dysfunction, ZEA caused significant declines in sperm quality, disruption of seminiferous tubules, and impaired structure of goat testicular interstitial supporting cells along with blood-testis barrier function. Concurrently, supporting cells exhibited numerous vacuoles and excessive endoplasmic reticulum swelling (25) (Figure 3).

#### 2.2.2 Genotoxicity

Extensive research indicates that ZEA exhibits genotoxicity, capable of inducing chromosomal aberrations, DNA damage, and gene mutations (26). In aquatic models, treating zebrafish embryos with 350–950 μg/L ZEA until 96 h post-fertilization (hpf) resulted in a highly significant increase in comet assay olive tail moments (OTM). Extensive apoptotic cells appeared in brain regions, accompanied by oxidative stress and DNA damage, with injury severity positively correlated with ZEA concentration and exposure duration (27). In rat primary testicular supporting cells, ZEA induced DNA damage and inhibited cell proliferation by activating the P-ATM → p53 → p21 signaling pathway, revealing its detrimental effects on male germ cell genetic material (28). Furthermore, in human breast cancer MCF7 cells, ZEA significantly elevated 5-methylcytosine levels and upregulated the expression of metabolism-related genes such as DNMT1, MGMT, IGF1, and HK2. However, it had no significant effect on DNA methylation or related gene expression in human normal breast epithelial MCF10F cells, suggesting that its genotoxicity exhibits cell specificity (29).

#### 2.2.3 Immunotoxicity

The vast majority of immune cells express estrogen receptors on their surfaces. When ZEA binds to these receptors, it disrupts the normal functioning of the body’s immune system. The immune system comprises three major components: immune molecules, immune cells, and immune organs. *In vitro* experiments with chicken spleen lymphocytes showed that after 48 h of exposure to 25 μg/mL ZEA, IL-2 mRNA levels increased while IL-6 and IFN-*γ* levels decreased significantly, indicating that ZEA infection may interfere with cytokine secretion in chicken spleen lymphocytes (30). Literature reports indicate that in experiments where ZEA induces porcine spleen damage, ZEA upregulates the expression and synthesis of pro-inflammatory cytokines such as TNF-*α* and IL-8, activates the JNK pathway, and inhibits p38/MAPK and NF-κB, thereby disrupting immune homeostasis (31). Liang et al. (32) found that ZEA significantly inhibited mouse thymic epithelial cell proliferation in a dose- and time-dependent manner. Furthermore, literature reports indicate that ZEA and its metabolites can also affect intestinal and humoral immunity, such as altering intestinal mucosal IgA levels and inhibiting lymphocyte proliferation, with metabolites often exhibiting greater toxicity than ZEA itself (33).

#### 2.2.4 Cytotoxicity

ZEA exerts cytotoxic effects on multiple cell types. It induces oxidative stress and activates apoptotic signaling pathways, leading to oxidative damage, apoptosis, or abnormal proliferation in various cells. In germ cells, ZEA upregulates ERS marker proteins such as GRP78, CHOP, and PERK in porcine endometrial epithelial cells (PECs), activates the JNK pathway, promotes *β*-catenin nuclear translocation, blocks G1 phase progression, and inhibits Bax and Caspase3, thereby inducing abnormal cell proliferation (34). In porcine trophoblast cells (pTr), ZEA activates the PERK-eIF2α-ATF4-CHOP and MAPK pathways, inhibits PI3K/AKT, elevates ROS and Ca^2+^, disrupts mitochondrial membrane potential, and induces autophagic apoptosis (35). In porcine endometrial stromal cells (ESCs), ZEA activates the ASK1-JNK pathway via the endoplasmic reticulum stress (ERS), elevates nuclear p-JNK, upregulates Bax and Caspase3/9 while downregulating Bcl-2, thereby intensifying apoptosis (36). In immune cells, 1–25 μg/mL ZEA inhibited LPS-activated mouse splenic lymphocyte proliferation with inhibition rates ranging from 5.62 to 88.17% over 24–72 h. It concurrently induced DNA fragmentation, with apoptosis rates increasing to 33.15% at higher concentrations (37).

#### 2.2.5 Hepatotoxicity and nephrotoxicity

As the primary organs for ZEA metabolism and excretion in the body, the liver and kidneys are key targets for its toxicity. Regarding hepatotoxicity, studies have demonstrated that ZEA treatment of primary support cells for 24 h promotes intracellular ROS production, reduces cell viability and antioxidant enzyme activity, and causes a decrease in mitochondrial membrane potential. Concurrently, it induces activation of the Caspase-dependent apoptosis pathway and enhances autophagy activity (38), indicating that its hepatotoxic mechanism is closely related to oxidative stress (39, 40). The kidney serves as a secondary metabolic organ and is one of the primary target organs for zearalenone. After multiple enterohepatic circulations *in vivo*, zearalenone is filtered by the kidneys and ultimately excreted in urine. Specific effects include inducing glomerular atrophy, tubular epithelial cell degeneration, and proteinuria. In renal toxicity studies, female Wistar rats administered 40 mg/kg ZEA via gavage for one week exhibited significantly reduced kidney weight, markedly elevated serum blood urea nitrogen (BUN) and uric acid (UA) levels, significantly decreased creatinine (CRE), along with increased malondialdehyde (MDA) in kidney tissue. Along with marked decreases in antioxidant enzyme activities such as superoxide dismutase (SOD), catalase (CAT), and glutathione peroxidase (GSH-px). Pathological lesions observed included renal vascular congestion, tubular dilatation, interstitial congestion, scattered cellular degeneration and necrosis, and glomerular cavity dilatation (41). Further mechanistic studies revealed that in porcine kidney epithelial cells (PK-15), ZEA induces apoptosis by activating ROS-mediated mitochondrial apoptosis pathways, upregulating pro-apoptotic proteins such as Bax and Caspase-3, and downregulating the anti-apoptotic protein Bcl-2 (42). Moreover, after ZEA enters the animal body, the liver can metabolize this toxin into two isomers: *α*-zearalenol (α-ZOL) and *β*-zearalenol (β-ZOL). The metabolite α-Zearalenol (α-ZOL) exhibits even greater cellular toxicity than ZEA itself, suggesting its metabolic activation process plays a critical role in liver and kidney toxicity (43).

### 2.3 Species-specific characteristics of ZEA toxicity

ZEA is a common mycotoxin that exerts toxic effects on various animal species. Pigs, chickens, cattle, and other animals are exposed to this toxin through consumption of ZEA-contaminated feed. Significant differences exist in ZEA sensitivity across species, primarily manifested in exposure routes, toxic doses, and typical clinical symptoms. Pigs are the most susceptible species to ZEA. Even trace amounts of ZEA in feed—as low as 0.1–0.15 mg/kg—can induce reproductive tract inflammation in sows. The maximum allowable levels of ZEA in feed for piglets and growing-finishing pigs are 0.1 mg/kg and 0.5 mg/kg, respectively. Typical clinical symptoms in pigs are concentrated in the reproductive system. For example, weaned gilts may exhibit vulvar hypertrophy and ovarian atrophy, while adult sows may experience infertility and pseudopregnancy. Boars may show testicular atrophy, sperm abnormalities, and reduced libido (4). In contrast, poultry and ruminants demonstrate higher tolerance. High ZEA doses (≥5 mg/kg) impair laying hen production performance and disrupt reproductive hormone secretion. At ZEA levels exceeding 5 mg/kg, laying hens exhibit significantly reduced average egg weight, markedly decreased blood luteinizing hormone (LH) levels, and markedly elevated progesterone levels, with these changes exhibiting a dose-dependent effect (44). Cattle, however, possess rumen microorganisms that partially degrade ZEA, resulting in a higher toxic threshold and atypical clinical symptoms. The primary potential impact is a slight reduction in reproductive performance. Case reports indicate that when corn zearalenone levels in feed reach 200 mg/kg, growing cattle exhibit toxic symptoms such as restlessness, reddened vaginal mucosa, and swollen labia (45).

These differences primarily stem from interspecies variations in metabolic pathways: pigs convert ZEA into the more toxic *α*-zearalenol, whereas poultry and ruminants produce less toxic metabolites. Therefore, subsequent research on natural product detoxification strategies for ZEA must account for species specificity: For swine, focus should be placed on developing formulations that block ZEA’s estrogen-like activity and promote excretion. For poultry and cattle, emphasis should shift toward additives that protect intestinal health and enhance overall production performance, enabling precise and effective detoxification interventions.

## 3 Research on natural products for ZEA attenuation

### 3.1 Plant-derived natural products

#### 3.1.1 Flavonoids

Upon entering the animal body, ZEA can cause structural damage and functional impairment in multiple organs and tissues through various pathways, including oxidative stress, apoptosis, inflammatory responses, and endocrine disruption. In recent years, flavonoid natural medicines have garnered significant attention in ZEA poisoning research due to their wide availability, low toxicity, and multifaceted biological activities. Their detoxifying effects primarily achieve ZEA poisoning mitigation through multi-pathway, multi-target interventions.

Regarding the alleviation of oxidative damage, ZEA induces the production of large amounts of reactive oxygen species (ROS) upon entering the body. When ROS generation exceeds clearance, it triggers lipid peroxidation reactions, producing harmful substances such as malondialdehyde (MDA). Concurrently, it inhibits the activity of antioxidant enzymes like superoxide dismutase (SOD) and glutathione peroxidase (GSH-Px), disrupting the body’s oxidative stress balance and causing oxidative damage to tissues like the uterus, liver, and spleen. Flavonoid-based natural medicines such as hyperoside, quercetin, rutin, and silymarin effectively address this issue. On one hand, they directly react with ROS to eliminate them, reducing ROS attacks on cells. On the other hand, they activate the body’s own antioxidant enzyme system, enhancing the activity and expression levels of antioxidants like SOD and GSH-Px, thereby strengthening the body’s ROS clearance capacity. Through these dual mechanisms, flavonoids significantly reduce the production of lipid peroxidation byproducts like malondialdehyde (MDA), maintain oxidative stress equilibrium, and mitigate ZEA-induced oxidative damage across multiple tissues and organs. These effects have been validated in various animal models and cellular experiments (46–51).

In inhibiting apoptosis and inflammatory responses, ZEA causes harm to the body by activating the apoptosis pathway. ZEA promotes the activation of Caspase family proteins while regulating the expression of apoptosis-related proteins, leading to the upregulation of pro-apoptotic protein Bax and the downregulation of anti-apoptotic protein Bcl-2. This disrupts the balance of apoptosis, resulting in massive cell death in tissues. Additionally, ZEA induces the release of inflammatory mediators such as tumor necrosis factor-*α* (TNF-α) and interleukin-6 (IL-6), activating inflammatory signaling pathways like nuclear factor-κB (NF-κB) to trigger inflammatory damage. Flavonoid compounds like rutin and baicalin exhibit significant inhibitory effects against these mechanisms. Studies indicate that these compounds restore apoptotic balance by downregulating apoptosis-related proteins like Bax and Caspase-3 while upregulating Bcl-2 expression, thereby inhibiting excessive cell death (52, 53). For example, in a chick liver injury model established with 2.5 mg/kg ZEA, chicks were simultaneously administered 20 mg/kg, 40 mg/kg, or 80 mg/kg baicalin via oral gavage for one week. Immunohistochemical analysis revealed that baicalin dose-dependently reduced ZEA-induced hepatocyte apoptosis in chicks. Specifically, the 80 mg/kg baicalin group exhibited no significant difference in hepatocyte apoptosis compared to the blank control group, with virtually no discernible hepatocyte apoptosis observed (Xu Jingnan, 2022). In a ZEA-induced porcine endometrial stromal cell injury model, rutin was demonstrated to further enhance apoptosis inhibition by activating the nuclear factor E2-related factor 2 (Nrf2) signaling pathway (54). Treatment of porcine ESCs with 52.03 μM ZEA significantly increased the apoptosis rate. The addition of 25 μM rutin significantly reduced ZEA-induced apoptosis in porcine ESCs (*p* < 0.01). When Nrf2 was pre-silenced (si-Nrf2), ZEA-induced apoptosis further increased, and the addition of 25 μM rutin significantly suppressed this enhanced apoptotic effect (Z + N + R group vs. Z + N group, p < 0.01) (Chen et al., 2025). Concurrently, these flavonoids inhibit the activation of inflammatory signaling pathways such as NF-κB, reducing the secretion of inflammatory mediators like TNF-*α* and IL-6. This effectively alleviates inflammatory states in tissues like the liver and mitigates ZEA-induced inflammatory damage (55). In a model inducing porcine renal epithelial cell damage, quercetin targets CaSR to inhibit the CaSR/CaMKII pathway, regulating calcium homeostasis and maintaining mitochondrial dynamics stability, thereby preventing ZEA-induced apoptosis (56).

Regarding antagonizing endocrine-disrupting effects, ZEA exhibits estrogen-like activity by competitively binding to estrogen receptor alpha (ERα) and estrogen receptor beta (ERβ) in animals. This interferes with normal hormonal signaling pathways, leading to reproductive endocrine disorders and subsequent reproductive system abnormalities in animals, such as abnormal follicle development in sows and reduced sperm quality in boars. Flavonoid compounds like soy isoflavones demonstrate unique advantages in this context. They compete with ZEA for estrogen receptor binding sites. Due to their stronger binding affinity, they reduce ZEA’s receptor occupancy, thereby diminishing its estrogenic effects. Simultaneously, soy isoflavones regulate estrogen receptor expression levels, restoring normal hormone signaling pathways and maintaining reproductive endocrine balance (57). Furthermore, Practical application studies reveal that supplementing sow diets with soy isoflavones not only antagonizes ZEA’s estrogenic effects but may also accelerate the biotransformation and degradation of ZEA and its metabolites within the body by regulating the activity of relevant metabolic enzymes in tissues such as the liver. This approach reduces ZEA residues in the liver and muscle tissues of gilts during puberty and post-puberty, thereby mitigating the persistent harm caused by ZEA to animal organisms (58).

In summary, flavonoid-based natural medicines significantly mitigate ZEA toxicity through multiple mechanisms, including alleviating oxidative damage, inhibiting apoptosis and inflammatory responses, and antagonizing endocrine disruption. As research continues to advance, the application prospects of flavonoid-based natural medicines in alleviating ZEA poisoning will become increasingly broad, providing important research directions and potential solutions for addressing livestock production and food safety issues caused by ZEA contamination.

#### 3.1.2 Polysaccharides

Polysaccharide-based natural medicines, characterized by their wide availability, high biocompatibility, minimal toxicity, and multifaceted biological activities, including antioxidant, immunomodulatory, and anti-inflammatory effects, have emerged as a significant research focus for alleviating ZEA poisoning. These substances exert protective effects through multidimensional and multitranslational pathways by precisely targeting key pathological stages of ZEA poisoning, offering novel strategies for its prevention and control.

Polysaccharides from diverse sources demonstrate unique advantages in mitigating ZEA-induced oxidative stress and apoptosis. *Pteridium aquilinum* polysaccharide (PAP-1b) exhibited significant protective effects in studies targeting testicular supporting cells of Changbai pigs (59). As critical cells maintaining spermatogenesis, testicular supporting cells are highly susceptible to ZEA toxicity. PAP-1b enhances cellular ROS scavenging capacity by boosting the activity of antioxidant enzymes such as superoxide dismutase (SOD) and glutathione peroxidase (GSH-Px). Concurrently, it reduces levels of the lipid peroxidation product malondialdehyde (MDA) and the cellular damage marker lactate dehydrogenase (LDH), effectively mitigating ZEA-induced oxidative damage. Further studies revealed that PAP-1b also modulates apoptosis-related molecules to restore the Bax/Bcl-2 ratio and mitochondrial membrane potential, thereby inhibiting apoptosis. Its protective effects are closely associated with the PI3K-Akt signaling pathway and glutathione metabolism pathway. Key molecules in these pathways, GPX1 and SELENOK, play central roles in mediating PAP-1b’s detoxification effects. For example, a porcine testicular supporting cell damage model was established using 100 μM ZEA. After 4 h of pretreatment with 150 μg/mL Pteridium polysaccharide (PAP-1b), cells were cultured continuously for 48 h. flow cytometry analysis revealed significantly elevated apoptosis rates in the ZEA-treated group alone, whereas the PAP-1b-treated group exhibited significantly reduced apoptosis rates compared to the ZEA group (*p* < 0.05), with apoptosis levels approaching those of the blank control group (Shi et al., 2025).

Regarding the repair of ZEA-induced immune suppression, the thymus, as a central immune organ, is a critical site for T lymphocyte differentiation and maturation. ZEA can impair immune function by inducing thymocyte apoptosis. Astragalus polysaccharides (APS) significantly protect chicken thymocytes by downregulating the expression of endoplasmic reticulum stress-related genes such as ATF4, ATF6, and CRP78, as well as pro-apoptotic genes like Bax and Caspase-3, while simultaneously upregulating the expression of the anti-apoptotic gene Bcl-2, thereby inhibiting ZEA-induced thymocyte apoptosis. At a concentration of 200 μg/mL, APS exhibited optimal protective effects on thymocytes, effectively restoring thymic tissue morphology, enhancing immune cell activity, repairing ZEA-induced immune suppression, and strengthening the body’s antitoxic capacity (60).

Beyond the aforementioned polysaccharides, substances such as Poria cocos polysaccharide (PCP), *Lycium barbarum* polysaccharide (LBP), and selenium-chitosan also demonstrate promising potential in alleviating ZEA toxicity (61–63). PCP alleviates ZEA-induced oxidative damage across multiple tissues by elevating antioxidant enzyme activity (SOD, GPx) in mouse liver and kidney tissues, reducing MDA levels, and maintaining normal organ physiological functions. LBP specifically protects against ZEA-induced renal injury by inhibiting ZEA-induced mitochondrial apoptosis and autophagy in mouse kidneys. It reduces pathological damage and fibrosis in renal tissue by downregulating pro-apoptotic and autophagy-related molecules, thereby preserving renal filtration and metabolic functions. Selenium-chitosan, a conjugate of selenium and chitosan, demonstrated superior protective effects in a porcine endometrial epithelial cell injury model. It significantly downregulates ZEA-induced expression of genes, including JNK, ASK1, c-Jun, MKK4, and p53 by reducing intracellular ROS levels. By modulating the JNK/SAPK signaling pathway, it effectively mitigates ZEA-induced cell cycle arrest, mitochondrial damage, and apoptosis, offering a novel strategy for protecting endometrial function and reducing reproductive disorders in livestock and poultry.

In summary, polysaccharide-based natural medicines exert detoxification effects through multiple pathways, including enhancing antioxidant capacity, precisely regulating signaling pathways, and inhibiting apoptosis. Their mechanisms of action cover several key pathological stages of ZEA poisoning. These findings not only enrich the theoretical framework for ZEA poisoning prevention and control but also provide crucial experimental evidence for developing safe and effective natural anti-ZEA toxins. This research holds significant practical and applied value for promoting the healthy development of livestock and poultry farming and ensuring food safety.

#### 3.1.3 Polyphenols

ZEA is a common mycotoxin that damages the liver, reproductive system, and intestines. Polyphenolic natural medicines possess antioxidant, anti-inflammatory, and cell signaling pathway-modulating properties, enabling detoxification through multiple pathways. Proanthocyanidins (PCs) are internationally recognized as potent natural antioxidants and are widely used in ZEA detoxification research. Their protective effects primarily arise from regulating pathways associated with oxidative stress and apoptosis. In the mouse intestinal epithelial cell (MODE-K) model, ZEA activates the endoplasmic reticulum stress (ERS)-apoptosis pathway by upregulating ERS-related mRNA and protein expression, including CHOP, GRP78, JNK, and Caspase-12. Simultaneously reducing anti-apoptotic protein Bcl-2 levels and increasing pro-apoptotic protein Bax levels to induce apoptosis. It also inhibits superoxide dismutase (SOD) and glutathione peroxidase (GSH-Px) activity, decreases glutathione (GSH) content, and increases malondialdehyde (MDA) production, leading to oxidative damage. Conversely, PCs at concentrations of 5–15 μg/mL significantly reversed these changes by reducing apoptosis rates, restoring antioxidant enzyme activity and GSH content, inhibiting MDA elevation, and downregulating the expression of molecules associated with the ERS apoptosis pathway. This mechanism involves suppressing ERS-induced apoptosis pathways and alleviating oxidative stress (64). In mouse testicular supporting cells (TM4 cells), ZEA disrupts the Nrf2/ARE signaling pathway, downregulating mRNA and protein expression of Nrf2 and its downstream target genes HO-1, NQO1, GSH-Px, and *γ*-GCS, thereby weakening cellular antioxidant capacity. PCs (2.5–10 μg/mL) can activate the Nrf2/ARE pathway, upregulate the expression of the aforementioned antioxidant genes and proteins, enhance SOD and GSH-Px activity, reduce MDA accumulation, and decrease lactate dehydrogenase (LDH) release, thereby mitigating ZEA-induced cellular oxidative damage and apoptosis. The protective effect is optimal at a concentration of 5 μg/mL (65).

Curcumin (CUR), an extract from the ginger family, exhibits antioxidant, anti-inflammatory, and gut microbiota-modulating effects, demonstrating protective actions against ZEA-induced damage to the liver, kidneys, and reproductive system. In a mouse liver injury model, ZEA (40 mg/kg) induced hepatocyte edema, mitochondrial vacuolation, elevated serum AST and ALT activity, increased ROS and MDA levels in liver tissue, decreased SOD, CAT, and GSH-Px activity, activated the NLRP3 inflammasome, and upregulated NLRP3, Caspase-1p20 protein, and IL-1β expression. Conversely, 150 mg/kg curcumin mitigated hepatic pathological damage through dual antioxidant and anti-inflammatory actions, restoring antioxidant enzyme activity, reducing ROS and MDA levels, inhibiting NLRP3 inflammasome activation and IL-1β release, and alleviating hepatic oxidative stress and inflammatory responses (66). In porcine kidney epithelial cells (PK-15 cells), ZEA (36.55 μg/mL) induces cellular oxidative stress, increases ROS and MDA production, and decreases SOD and CAT activity. Curcumin (6.25–25 μmol/L) activates the SIRT1/FOXO1 signaling pathway, upregulates SIRT1 protein expression, and reduces FOXO1 acetylation. This promotes mRNA and protein expression of downstream antioxidant enzymes Mn-SOD and CAT, scavenges ROS, reduces lipid peroxidation, and alleviates ZEA-induced oxidative damage in renal cells (42). Furthermore, in a male mouse model of reproductive damage, ZEA (40 mg/kg) disrupts gut microbiota balance by decreasing Lactobacillus abundance while increasing Prevotella and Bacteroides abundance. This activates the IL-17A-TNF-*α* signaling pathway in testes, reducing testosterone secretion, decreasing sperm survival rates, and increasing deformity rates. Curcumin at 200 mg/kg modulates gut microbiota structure, restores beneficial bacterial abundance, inhibits IL-17A pathway activation, elevates testosterone levels, and improves sperm quality. Its protective effects are associated with the regulation of the “gut microbiota-testicular axis” (67).

Resveratrol (RSV) is a natural polyphenolic antioxidant primarily found in plants such as grapes and *Polygonum cuspidatum*. RSV can reduce ZEA toxicity through multiple pathways. Regarding liver protection, in a ZEA-induced liver injury mouse model, RSV alleviated pathological damage, restored antioxidant enzyme activity, inhibited NF-κB nuclear translocation and inflammatory factor release, demonstrating optimal protective effects (68). In mouse testicular supporting cells, RSV activates the PI3K/Akt pathway, promotes Akt phosphorylation, thereby driving Nrf2 nuclear translocation and upregulating HO-1 expression. This enhances cellular antioxidant capacity, reduces ROS production, and inhibits apoptosis-related proteins (Caspase-3, PARP cleavage) while increasing the Bax/Bcl-2 ratio, thus preventing cell death (69). Regarding intestinal protection, ZEA disrupts the intestinal barrier in mice. A 100 mg/kg RSV dose restores intestinal structure and barrier function by activating the Nrf2 pathway and inhibiting the NF-κB pathway to alleviate damage (70).

In summary, polyphenolic natural compounds mitigate ZEA toxicity through multi-targeted, multi-pathway mechanisms, including regulating oxidative stress-related pathways, inhibiting inflammatory pathways, and improving gut microbiota dysbiosis. Different polyphenolic compounds exhibit distinct target profiles: proanthocyanidins and resveratrol primarily regulate intracellular antioxidant and apoptosis pathways, while curcumin combines antioxidant, anti-inflammatory, and gut microbiota-modulating effects. These findings provide theoretical foundations for ZEA poisoning prevention and treatment, laying the groundwork for polyphenolic applications in animal husbandry.

### 3.2 Microbial-derived natural products

#### 3.2.1 Bacillus species

Bacillus species demonstrate exceptional performance in mitigating zearalenone toxicity due to their potent degradation capabilities and environmental adaptability. For instance, the Bacillus paeiliosus strain PA26-7 efficiently degrades zearalenone by secreting extracellular enzymes. This strain degrades zearalenone under a wide range of conditions: initial medium pH 4.0–8.0 and cultivation temperatures 25–60 °C. The degradation products exhibit lower cytotoxicity and estrogenic activity compared to zearalenone (71). Additionally, the *Bacillus velezensis* strain B.26 is particularly noteworthy. Studies indicate that this strain can degrade 91.64% of ZEA within 24 h at 70 °C and pH 10.0 (72). This high degradation efficiency is primarily attributed to its secreted CotA laccase and Prx peroxidoreductase. Through genomic mining and molecular cloning techniques, researchers successfully isolated and recombinantly expressed these two enzymes. CotA enzyme achieved over 91% degradation of ZEA within 6 h at 70 °C and pH 8.0, with its activity significantly enhanced by adding ions such as Na^+^ and Cu^2+^. Prx enzyme reached a degradation rate of 59.74% for ZEA within 6 h at 70 °C and pH 11.0. This indicates that both enzymes exhibit high degradation efficiency under both high-temperature and alkaline conditions. Their degradation products are low-toxicity compounds C17H24O4 and C12H16O4, without generating high-risk estrogen metabolites such as *α*-ZEL, thereby ensuring the safety of the degradation process.

Bacillus degradation enzymes show broad application prospects for mitigating zearalenone toxicity. These enzymes not only demonstrate high degradation efficiency under laboratory conditions but are also considered to possess significant potential for application in the food and feed industries due to their environmental friendliness and safety. With further technological optimization and commercialization, these degradative enzymes are expected to become powerful tools for degrading and removing mycotoxin contamination. Additionally, the *Bacillus amyloliquefaciens* strain XJ-140 was screened for its high-efficiency ZEA degradation capability, degrading 93.75% of ZEA (2 μg/mL) within 24 h of incubation. This degradation primarily occurs via extracellular enzymes, supplemented by cell wall adsorption (73).

#### 3.2.2 Yeast

Yeast exhibits significant potential for degrading ZEA in corn. Recent studies have revealed unique mechanisms and high efficiency in ZEA degradation by various yeast strains. For instance, the Tibetan yeast strain *Saccharomyces cerevisiae* KAB68 demonstrates outstanding ZEA degradation capabilities. At a culture density of 1 × 10^8^ cells/mL, this strain achieved an 80.3% degradation rate for ZEA. Studies indicate that this yeast strain removes ZEA through a dual mechanism involving adsorption and intracellular biodegradation (74). This dual-action mechanism not only enhances degradation efficiency but also ensures the environmental friendliness of the degradation process.

The mechanism of yeast-mediated ZEA degradation primarily involves adsorption and biotransformation. During adsorption, functional groups on the yeast cell surface—such as O–H, N–H, C═O, and C–O—bind to ZEA molecules, thereby reducing their free concentration in solution (75). During biotransformation, yeast secretes specific enzymes like laccase and peroxidase to degrade ZEA into less toxic metabolites. For instance, Pichia pastoris has been engineered to express zearalenone-degrading enzymes, which efficiently break down ZEA during fermentation (76). Additionally, components of the yeast cell wall participate in the adsorption of ZEA, further enhancing its degradation capacity.

These studies not only elucidate the molecular mechanisms by which yeast degrades ZEA but also provide a theoretical basis for developing yeast-based bio-detoxifiers. Future research should focus on further optimizing degradation conditions for yeast to enhance its efficiency and stability in practical applications, as well as identifying additional yeast strains with high degradation capacity.

#### 3.2.3 Lactic acid bacteria

Lactic acid bacteria have garnered increasing attention in recent years for their role in degrading zearalenone (ZEA) due to their widespread application and safety profile in the food industry. Research indicates that lactic acid bacteria reduce ZEA toxicity through two primary mechanisms: adsorption and biotransformation. For instance, current research demonstrates that the novel lactic acid bacteria strain LBC-4 can degrade ZEA into less toxic derivatives, with optimal detoxification occurring at 37 °C and pH 7–8 (77). In animal studies, Lactobacillus significantly reduced ZEA’s toxic effects on rat blood, liver, kidneys, and uterus, while aiding in the restoration of normal physiological and biochemical parameters (78).

Although lactic acid bacteria show great potential for ZEA detoxification, further research is needed on their biodegradation mechanisms, the toxicity of degradation products, and microbial safety for animals.

### 3.3 Mineral-based natural products

Research on mineral-based natural products for reducing zearalenone toxicity has primarily focused on activated carbon and montmorillonite. As a natural mineral adsorbent, montmorillonite exhibits adsorption properties toward zearalenone. It can adsorb various mycotoxins, thereby reducing their levels in feed. First, most mineral adsorbents exhibit poor adsorption efficiency for ZEA. Experimental results indicate that the adsorption rate of sodium-based montmorillonite ZEA is only 4%, suggesting certain limitations in the physical adsorption method. This is attributed to ZEA’s weakly polar groups and low electrophilicity, making charge-based adsorption difficult (79). Second, when using montmorillonite as an adsorbent, desorption of zearalenone must be considered. Research indicates that montmorillonite exhibits good adsorption of zearalenone under acidic conditions at pH 3. However, desorption of adsorbed zearalenone occurs at both pH 3 and pH 5, with particularly pronounced desorption observed at pH 5 (80). This implies that in practical applications, relying solely on montmorillonite’s adsorption capacity may not fully eliminate zearalenone, necessitating comprehensive consideration of both adsorption and desorption phenomena. Additionally, activated carbon—a highly porous, insoluble powder with a large specific surface area—exhibits strong adsorption capacity but non-selectively binds feed nutrients, potentially causing adverse effects in animals. For instance, adding 2.5% or 5.0% montmorillonite to pig diets resulted in liver damage (81). Mineral-based natural products like montmorillonite and activated carbon show potential for reducing zearalenone toxicity, but limitations include restricted adsorption capacity, desorption issues, and indiscriminate nutrient binding. In production practice, the selection and optimization of adsorbent usage protocols should be based on factors such as feed pH, animal digestive physiology, and the type and concentration of zearalenone in the feed. This approach ensures more effective zearalenone removal, safeguarding feed safety and animal health.

### 3.4 Bee-derived natural products

ZEA primarily exhibits toxic effects including reproductive toxicity, immunosuppression, and liver damage. These effects not only disrupt normal physiological functions in animals but also lead to reduced livestock production efficiency and economic losses. Bee-derived natural products are substances formed through fermentation when bees collect raw materials such as plant pollen and nectar and combine them with their own secretions. Core categories include bee pollen, honey, and propolis, while other components with potential bioactivity encompass beeswax and royal jelly (82). Recent domestic and international studies confirm that bee-derived natural products are rich in bioactive substances such as polyphenols, flavonoids, polysaccharides, amino acids, vitamins, and minerals. They exhibit multiple pharmacological effects including antioxidant, anti-inflammatory, immunomodulatory, and hepatoprotective/nephroprotective properties (83, 84). This provides scientific rationale for their potential to antagonize ZEA toxicity and positions them as an emerging direction in ZEA detoxification research.

From the perspective of the correlation between chemical composition and pharmacological effects, the detoxification potential of bee-derived natural products is closely related to their unique composition. Bee pollen, a granular substance formed when bees collect pollen grains from plant anthers and mix them with their own secretions, contains core active components such as flavonoids (e.g., rutin, quercetin), polyphenols, and polysaccharides. Among these, flavonoids can scavenge excess reactive oxygen species (ROS), activate the antioxidant pathway via nuclear factor E2-related factor 2 (Nrf2), enhance the activity of antioxidant enzymes such as superoxide dismutase (SOD) and glutathione peroxidase (GSH-Px), and simultaneously inhibit the production of malondialdehyde (MDA), a lipid peroxidation product. This mechanism closely aligns with the previously described pathway by which plant-derived flavonoids (e.g., soy isoflavones) antagonize ZEA-induced oxidative damage. Regarding immune regulation, the polysaccharides in bee pollen promote T-lymphocyte and B-lymphocyte proliferation, elevate immunoglobulin (IgA, IgM) secretion levels, and enhance leukocyte activity, thereby repairing damage to immune organs. This is crucial for mitigating ZEA-induced immunosuppression. Research confirms that adding 2–3% bee pollen to broiler feed significantly alleviates thymus and spleen atrophy caused by ZEA (5 mg/kg) and reverses the decline in peripheral blood cytokine levels (e.g., IL-2, IFN-*γ*). This effect is mediated by activating the PI3K-Akt signaling pathway and suppressing excessive NF-κB inflammatory pathway activation, directly supporting the detoxification value of “bee pollen” highlighted in the keywords (84).

Honey, as the most extensively studied category among natural apicultural products, primarily consists of glucose and fructose (accounting for approximately 60–80%), alongside phenolic acids (such as caffeic acid and chlorogenic acid), flavonoids (including apigenin and kaempferol), and enzymes (like sucrase and catalase) (85, 86). These components confer honey with significant antioxidant and hepatoprotective activities: In a mouse model of alcoholic liver injury, 15 g/kg of red eucalyptus honey alleviated hepatic edema and inflammatory infiltration by upregulating mRNA and protein expression of CAT, SOD, and GSH-Px, while reducing serum alanine aminotransferase (ALT) and aspartate aminotransferase (AST) activity (85). In ZEA-induced liver injury, phenolic acids in honey mitigated hepatocyte apoptosis by inhibiting NLRP3 inflammasome activation, reducing proinflammatory factor release (e.g., IL-1β), and enhancing mitochondrial membrane potential stability. This mechanism synergized with curcumin’s antagonism of ZEA hepatotoxicity. Furthermore, in studies on boar semen preservation, adding 10% buckwheat honey increased SOD and CAT activity in semen, reduced ROS accumulation, and improved sperm motility and survival rate (86). This finding suggests honey may mitigate ZEA-induced damage to male germ cells through antioxidant pathways, providing direction for future research.

Propolis is a resinous substance collected by bees from plant buds and tree bark, processed by mixing with their own secretions. Its primary active components include flavonoids (e.g., morin, pinobanksin), terpenoids, and phenolic esters (e.g., phenethyl caffeate). Modern pharmacological studies confirm propolis exhibits broad-spectrum antibacterial, anti-inflammatory, and immune-enhancing effects: On one hand, its flavonoid compounds competitively bind estrogen receptors (ER*α*/ERβ), reducing ZEA’s binding efficiency to these receptors and thereby attenuating its estrogen-like effects—a mechanism similar to how soy isoflavones antagonize ZEA’s reproductive toxicity. On the other hand, phenethyl caffeate in propolis can inhibit JNK signaling pathway activation, reduce the release of pro-inflammatory factors TNF-α and IL-6, and repair ZEA-damaged immune organs. In broiler chicken trials, supplementing feed with 0.1–0.2% propolis significantly enhanced total antioxidant capacity (T-AOC) in ZEA-exposed chickens, promoted spleen lymphocyte proliferation and antibody production, and synergistically improved ZEA-induced immunosuppression with bee pollen (84).

Overall, bee-derived natural products alleviate ZEA toxicity through multiple mechanisms—including antioxidant, anti-inflammatory, immunomodulatory, and endocrine-disrupting effects—due to their multi-component, multi-target characteristics. These effects complement the detoxification mechanisms of plant- and microbe-derived natural products. However, current research has limitations: First, the mechanisms of bee-derived natural products are primarily studied at the whole-animal or cellular level, with their active components (e.g., bee pollen flavonoids, propolis terpenoids) and direct interactions with ZEA (e.g., molecular docking, enzyme activity regulation) yet to be fully elucidated. Second, bee product quality is significantly influenced by plant sources and harvesting seasons, lacking standardized extraction and application protocols. Future research should integrate metabolomics and proteomics to decipher the key bioactive components and signaling pathways through which bee-derived natural products antagonize ZEA toxicity. Concurrently, establishing quality control standards for bee-derived detoxifiers will provide scientific support for their industrial application in preventing and controlling ZEA contamination within the livestock industry.

## 4 Summary and prospects

ZEA contamination is a complex global issue in agriculture and food safety, posing a multi-organ, multi-dimensional threat to animal health. This review demonstrates that relying on a single method is insufficient for effective mitigation. Natural products, due to their diverse mechanisms of action, offer an ideal solution for building a multi-layered, comprehensive defense system against toxicity. From microbial enzymes that degrade ZEA at the source, to plant active compounds that antagonize its effects *in vivo*, and to polysaccharides and bee products that systemically enhance the body’s antioxidant and immune capacity, various natural strategies have their own focus and work synergistically.

Current research has progressed from simple efficacy observation to the exploration of molecular mechanisms, revealing the central role of key signaling pathways such as Nrf2, NF-κB, and ERS in the detoxification process. However, challenges remain: most studies are still at the experimental stage, far from large-scale clinical application; the safety and stability of microbial agents, the bioavailability of plant extracts, the selectivity of mineral adsorbents, and the standardization of bee products urgently need to be resolved.

Future research should concentrate on the following directions: First, deepen the investigation of the molecular mechanisms underlying natural product detoxification. Utilize modern biotechnologies such as gene editing and proteomics to further elucidate their target sites and signaling pathways, providing more precise targets for developing novel detoxifiers. Second, optimize the extraction, purification, and formulation processes of natural products to enhance their stability and bioavailability, developing natural detoxifier products suitable for different animal species and farming environments. Third, strengthen ecotoxicological research on microbial degradation of ZEA to assess its long-term impacts in natural environments, ensuring the safety and sustainability of microbial degradation technologies. Fourth, conduct large-scale field trials to validate the efficacy and safety of natural detoxifiers in practical aquaculture settings, advancing their industrial application. Additionally, international cooperation and exchange should be enhanced to integrate global resources in jointly addressing ZEA pollution, thereby contributing to food security and public health.
